# The 3-hydroxy-3-methylglutaryl-coenzyme A reductase inhibitors, simvastatin, lovastatin and mevastatin inhibit proliferation and invasion of melanoma cells

**DOI:** 10.1186/1471-2407-8-9

**Published:** 2008-01-16

**Authors:** Sharon A Glynn, Dermot O'Sullivan, Alex J Eustace, Martin Clynes, Norma O'Donovan

**Affiliations:** 1 National Institute for Cellular Biotechnology, Dublin City University, Glasnevin, Dublin 9, Ireland; 2 Cancer Prevention Fellow, Laboratory of Human Carcinogenesis, Center for Cancer Research, National Cancer Institute, Room 3044, 37 Convent Drive, Bethesda, MD 20892, USA

## Abstract

**Background:**

A number of recent studies have suggested that cancer incidence rates may be lower in patients receiving statin treatment for hypercholesterolemia. We examined the effects of statin drugs on *in vitro *proliferation, migration and invasion of melanoma cells.

**Methods:**

The ability of lovastatin, mevastatin and simvastatin to inhibit the melanoma cell proliferation was examined using cytotoxicity and apoptosis assays. Effects on cell migration and invasion were assessed using transwell invasion and migration chambers. Hypothesis testing was performed using 1-way ANOVA, and Student's t-test.

**Results:**

Lovastatin, mevastatin and simvastatin inhibited the growth, cell migration and invasion of HT144, M14 and SK-MEL-28 melanoma cells. The concentrations required to inhibit proliferation of melanoma cells (0.8–2.1 μM) have previously been achieved in a phase I clinical trial of lovastatin in patients with solid tumours, (45 mg/kg/day resulted in peak plasma concentrations of approximately 3.9 μM).

**Conclusion:**

Our results suggest that statin treatment is unlikely to prevent melanoma development at standard doses. However, higher doses of statins may have a role to play in adjuvant therapy by inhibiting growth and invasion of melanoma cells.

## Background

The statins are group of drugs routinely used in the treatment of lipid disorders, including hypercholesterolemia. Statins exert their effects through the inhibition of 3-hydroxy-3-methylglutaryl-coenzyme A reductase (HMG-CoA reductase). HMG-CoA reductase catalyses the conversion of HMG-CoA into mevalonate in the mevalonate biosynthetic pathway [1,2].

In addition to the cholesterol lowering effects of statins, a number of recent studies suggest that the cancer incidence rates may be lower in patients receiving statins. The Air Force/Texas Coronary Atherosclerosis Prevention Study (AFCAPS/TexCAPS) trial to evaluate the efficacy of lovastatin for preventing coronary events, found a significantly decreased incidence of new melanomas in the lovastatin arm compared with the placebo arm. In addition, among the 41 participants who developed melanoma, there was a trend, although not statistically significant, toward earlier stage at diagnosis in the lovastatin group [3]. A recent meta-analysis described the incidence of melanoma in 12 qualifying randomized controlled statin trials, with a total of 39,426 participants. They reported that a total of 127 melanomas occurred, 59 among the 19,872 statin group participants and 68 among the 19,554 control group participants. The reported odds ratio for melanoma was in the direction of a protective effect, but did not reach significance (OR = 0.87, 95% CI = 0.61 to 1.23). As the number of incident melanoma cases was low, a specific study to address the role of statins in preventing melanoma needs to be designed, with the required power to provide answers [4].

Lung cancer incidences rates in statin users were found to be significantly reduced (55% risk reduction) in the Veterans Affairs (VA) Health Care System case control study [5]. Several studies have also suggested that colorectal cancer rates are reduced amongst statin users, including the Molecular Epidemiology of Colorectal Cancer study [6], and in the Rhine-Neckar-Odenwald population case-control study [7]. Graaf *et al *[8] also reported that statin use was associated with a risk reduction of cancer of 20% (adjusted odds ratio [OR], 0.80; 95% CI, 0.66 to 0.96). However, other studies have shown no benefit for statin use with respect to cancer prevention. Jacob *et al *[9] examined the association between use of cholesterol-lowering drugs and colorectal cancer incidence among 132,136 men and women in the Cancer Prevention Study II Nutrition Cohort. Current or 5 year use of cholesterol-lowering drugs was not associated with colorectal cancer incidence. A recent meta-analysis study including 6662 incident cancers and 2407 cancer deaths showed that statins did not reduce the incidence of cancer (OR, 1.02; 95% CI, 0.97–1.07) or cancer deaths (OR, 1.01; 95% CI, 0.93–1.09) and there was no reduction in any individual cancer type [10]. These conflicting reports highlight the need for further studies on the effects of statins on cancer cells.

A number of studies have investigated the effects of statins on melanoma cells. Lovastatin induces apoptosis in A375 melanoma cells [11] and also enhances response to chemotherapy drugs in the B16 mouse model of melanoma [12,13]. Collisson *et al *[14] showed that atorvastatin inhibited *in vitro *invasion and *in vivo *metastasis of A375M melanoma cells. Depasquale and Wheatley [15] recently showed that lovastatin reduced both melanoma cell growth and angiogenesis in an *in vitro *co-culture model angiogenesis system. These studies support the hypothesis that statins may play a role in melanoma prevention or treatment. However, the conflicting evidence from the chemoprevention studies suggests that further investigation is required to determine if standard choletsterol-lowering doses of statins will inhibit the growth of melanoma cells.

In this study we compared sensitivity to simvastatin, lovastatin and mevastatin across a panel of melanoma, lung and breast cancer cell lines and systematically examined the effects of the statins on apoptosis, adhesion, motility and invasion in melanoma cells.

## Methods

### Chemicals

Pravastatin sodium, mevastatin sodium, lovastatin sodium and simvastatin sodium (activated forms) were obtained from Merck Biosciences Ltd (Nottingham, UK) and reconstituted in DMSO. All media, serum and other chemicals were obtained from Sigma (Dublin, Ireland), unless otherwise stated.

### Cell lines

DLRP and MCF-7 were cultured in ATCC media supplemented with 2 mM L-glutamine (Gibco) and 10% foetal calf serum. SK-MEL-28, M14, HT144, SKBR-3 and MDA-MB-453 were cultured in RPMI 1640 media supplemented with 2 mM L-glutamine (Gibco) and 10% foetal calf serum. The melanoma cell lines, SK-MEL-28 and M14 were obtained from the Division of Cancer Treatment and Diagnosis Tumor Repository, NCI. H1299 was cultured in RPMI 1640 media supplemented with 5% foetal calf serum. BT474A, a clonal population of BT474, was cultured in RPMI 1640 media supplemented with 4 mM L-glutamine (Gibco), 0.1 mg/ml bovine insulin and 10% foetal calf serum. Cells were maintained at 37°C. Antibiotics were not used in the growth media. All cell lines were free from mycoplasma as tested with the indirect Hoechst DNA staining method.

### Cytotoxicity assays

Sensitivity to pravastatin sodium, mevastatin sodium, lovastatin sodium and simvastatin sodium in the panel of cell lines was determined by the acid phosphatase method, which measures viable cell number after 6 days of continuous exposure to drugs as previously described [16]. Toxicity assays were performed on at least two separate occasions, in triplicate (3 individual 96 well plates) for each cell line for each drug. Eight replicate wells, on the 96 well plate were used per drug concentration.

### Apoptosis assay

The ability of the statins to induce apoptosis was assessed in M14 cells using the Guava Nexin kit (Guava Technologies, Hayward, CA). Briefly, 2.5 × 10^5 ^cells were plated per well in 24 well plates and incubated overnight to allow attachment. Cells were treated with lovastatin, mevastatin or simvastatin (4 and 8 μM) for 72 hours. 0.1 μM taxol was used as a control for induction of apoptosis. After 72 hours apoptotic cells in the medium were collected and adherent cells were trypsinized. The resulting pool of cells was centrifuged at 300×g for 5 minutes, then resuspended in 50 μl of fresh medium and transferred to a round-bottom 96 well plate (Costar). 150 μl of Nexin Working Reagent (10 μl Annexin V-PE, 5 μl Nexin 7-AAD and 135 μl 1× Nexin buffer) was added to each well and the plate was incubated protected from light for 20 minutes at room temperature. After incubation the samples were acquired on the Guava EasyCyte. The Annexin V-PE detects phosphatidyl-serine on the external membrane of apoptotic cells and 7-AAD, a cell impermeant dye, is an indicator of membrane structural integrity. 7-AAD is excluded from live, healthy cells and early apoptotic cells, but permeates late-stage apoptotic and dead cells. Cells which stain positive for Annexin V-PE and negative for 7-AAD are classified as early apoptotic cells, whilst cells which are positive for both represent late apoptotic cells.

### Invasion assays and motility assays

Invasion assays were performed by a modification of the method described by Albini *et al *[17,18]. Matrigel (Sigma) was diluted to 1 mg/ml in serum-free DMEM medium. 100 μl of 1 mg/ml matrigel was placed into each insert (Falcon) (8.0 mm pore size), in a 24-well plate (Costar). The inserts were incubated at 37°C for 1 hour to allow gel polymerisation. Cells were harvested and suspended in DMEM containing 10% FCS at a concentration of 1 × 10^6 ^cells/ml. The inserts were washed with the appropriate serum-free medium, then 100 μl of the cell suspension and 100 μl of statin at 2× concentration was added to each insert and 500 μl of appropriate medium containing 10% FCS was added to the well underneath the insert. Cells were incubated at 37°C for 24 hours. After this time, the inner side of the insert was wiped with a wet swab to remove the cells while the outer side of the insert was gently rinsed with PBS and stained with 0.25% crystal violet for 10 min, rinsed again and then allowed to dry. The inserts were then viewed under the microscope and the number of cells per field in 10 random fields, were counted at 200× magnification. The average number of cells per field was then multiplied by a factor of 140 (growth area of membrane/field area viewed at 200× magnification (calibrated using a microscope graticule)) to determine the total number of invading/migrating cells. The procedure for carrying out motility assays was identical to the procedure used for invasion assays with the exception that the inserts were not coated with matrigel.

### Integrin-mediated binding assays

Integrin mediated adhesion was examined using the α/β Integrin-mediated cell adhesion array combo kits (Chemicon, UK). These kits use mouse monoclonal antibodies generated against human alpha (α1, α2, α3, α4, α5, αV, and αvβ3), and beta (β1, β2, β3, β4, β6, αVβ5, and α5β1) integrins/subunits, that are immobilized onto a goat anti-mouse antibody coated micro-titre plate. The plate is then used to capture cells expressing these integrins on their cell surface. The strips were re-hydrated with 200 μL of PBS per well for 10 min at room temperature. The plate was tapped on tissue paper to remove PBS. SK-MEL-28 and HT144 cells were incubated in the presence or absence of 0.8 μM simvastatin for 24 hours. Cells were harvested and plated at 1 × 10^5 ^cells per well in triplicate in serum free culture medium and incubated at 37°C for 1 hour. Medium and unbound cells were removed from the wells and rinsed gently twice with 200 μl assay buffer. 100 μl of cell stain solution was added to each well and incubated for 5 minutes at room temperature. The plates were washed 3 times with de-ionized water and allowed to air dry. 100 μL of Extraction Buffer was added to each well and the plate read in an ELISA reader at 560 nm.

### Extracellular matrix (ECM) protein binding assays

Adhesion assays were performed on CytoMatrix™ screening kits (Chemicon, UK). 96-well plates pre-coated individually with fibronectin, vitronectin, laminin, collagen I or collagen IV were re-hydrated with 200 μL of PBS per well for 15 min at room temperature, then the PBS was removed. A control non-coated plate was included. SK-MEL-28 and HT144 cells were incubated in the presence or absence of 0.8 μM simvastatin for 24 hours. Cells were harvested and plated at 1 × 10^5 ^cells per well (replicates of seven) in serum free culture medium and incubated at 37°C for 1 hour. Medium and unbound cells were removed from the wells and rinsed gently with PBS. 100 μl of freshly prepared phosphatase substrate (10 mM *p*-nitrophenol phosphate in 0.1 M sodium acetate, 0.1% triton ×-100, pH 5.5) was added to each well. The plates were then incubated in the dark at 37°C for 2 h. The enzymatic reaction was stopped by the addition of 50 μl of 1 N NaOH. The optical density was determined at a wavelength of 405 nm, with a reference wavelength of 620 nm. The optical density at 620 nm was subtracted from that at 405 nm to give the appropriate optical density of each sample.

### Statistical analysis

Statistical analyses were performed using Stata Intercooled 9.0 and GraphPad Prism Version 4. A p-value < 0.05 was deemed significant. A p-value < 0.005 was deemed highly significant.

## Results

### Lung cancer and melanoma cell lines are sensitive to statins, while breast cancer cell lines are resistant

As statins have been proposed as chemoprevention agents for a variety of cancer types including lung cancer, breast cancer and melanoma, we tested a range of cancer cell lines for sensitivity to four statin drugs, simvastatin, lovastatin, mevastatin and pravastatin. Pravastatin failed to inhibit proliferation (data not shown). Of the three remaining drugs, simvastatin displayed the greatest potency, followed by lovastatin and finally mevastatin (Table 1). The cell lines were derived from lung carcinomas (H1299, DLRP), breast carcinomas (MCF-7, BT474A, MDA-MB-453, SKBR-3) and malignant melanomas (HT144, M14, SK-MEL-28). There was a statistically significant difference in statin resistance across all the groups (one-way Anova: p = 0.011 [lovastatin]; p = 0.028 [mevastatin]; p = 0.013 [simvastatin]), demonstrating variance in resistance to statins by cancer site. Further analysis showed that the breast cancer cell lines were more resistant to statins, when compared to the lung cancer and melanoma cell lines (2-sided unpaired Student's t-test, p = 0.002 (lovastatin), p = 0.025 (mevastatin), p = 0.016 (simvastatin)). The melanoma cell lines displayed the greatest sensitivity to the statins, although not statistically significantly lower than the lung cancer cell lines.

**Table 1:** IC_50 _values for lovastatin, mevastatin and simvastatin (± std. deviation)

**Cell Line**	**IC_50 _Lovastatin (μM)**	**IC_50 _Mevastatin (μM)**	**IC_50 _Simvastatin (μM)**
**Lung cancer**			
DLRP	1.7 ± 0.5	1.9 ± 0.1	0.9 ± 0.1
H1299	2.6 ± 0.4	5.1 ± 0.3	1.3 ± 0.2
**Melanoma**			
HT144	1.7 ± 0.12	2.0 ± 0.33	1.0 ± 0.10
M14	1.2 ± 0.05	1.9 ± 0.10	0.8 ± 0.05
SK-MEL-28	1.3 ± 0.15	2.1 ± 0.11	0.8 ± 0.11
**Breast cancer**			
BT474A	5.3 ± 0.8	11.1 ± 1.5	4.2 ± 1.8
SKBR-3	4.7 ± 1.6	4.3 ± 0.6	2.2 ± 0.3
MDA-MB-453	7.8 ± 1.2	12.2 ± 3.5	5.4 ± 1.0
BT-20	3.9 ± 2.5	4.8 ± 1.6	1.7 ± 0.7

### Statins induce apoptosis in M14 melanoma cells

**Figure 1:**
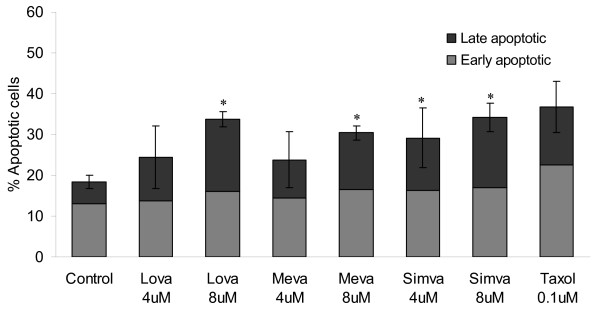
Induction of apoptosis in M14 melanoma cells by statins. M14 cells were exposed to statin treatment for 72 hours. Taxol (0.1 μM) was used as a positive control for apoptosis induction. Apoptosis was measured using an annexin V assay on the Guava EasyCyte, and results presented as % early and late apoptotic cells. (Lova = Lovastatin, Meva = Mevastatin, Simva = Simvastatin). Error bars represent std error of the mean for triplicate experiments. * indicates p < 0.05 using the Student's t-test to compare levels of late apoptosis in statin treated cells versus control.

To determine if the effects of the statins on proliferation were due to cytostatic or cytocidal effects on the melanoma cells we examined the ability of statins to induce apoptosis in the M14 cell line. M14 cells were exposed to two doses (4 and 8 μM) of lovastatin, mevastatin and simvastatin over 72 hours. Following statin exposure, the percentage of apoptotic cells was determined using an annexin V assay on the Guava EasyCyte. Apoptosis was increased in a dose-dependant manner in response to all three statins (Figure 1). Regression analysis shows that there is a significant trend toward increased levels of late apoptosis and total apoptosis with increasing statin concentration (Linear regression: p = 0.012 (r = 0.79) and p = 0.019 (r = 0.75) (late and total apoptosis for lovastatin), p = 0.003 (r = 0.86) and p = 0.044 (r = 0.46) (late and total apoptosis for mevastatin), p = 0.014 (r = 0.77) and p = 0.038 (r = 0.69) (late and total apoptosis for simvastatin)). No significant trend was observed for early apoptosis.

### Effect of simvastatin, lovastatin and mevastatin on melanoma cell migration and invasion

**Figure 2:**
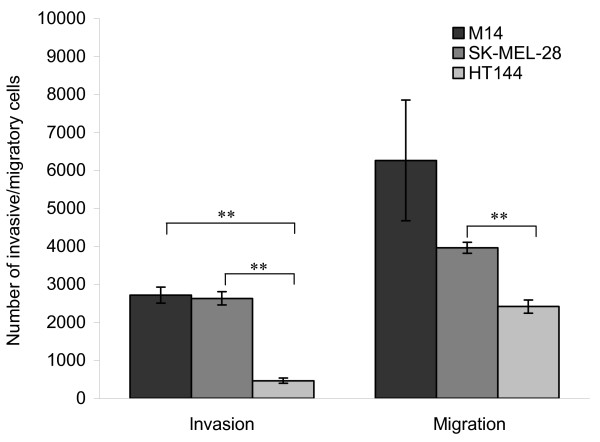
Comparison of invasion and migration potential of three melanoma cell lines. 1 × 10^5 ^cells were seeded in matrigel coated chambers for invasion assays and uncoated chambers for migration assays. Error bars represent the standard error of the mean for triplicate experiments. * indicates p < 0.05 and ** indicates p < 0.005 using the Student's t-test to compare levels of invasion and migration between cell lines.

The effects of the statins on cell migration and invasion were examined in three melanoma cell lines (M14, HT144 and SK-MEL-28), which were more sensitive to the statins than the lung and breast cancer cell lines. The three melanoma cell lines were both invasive and motile to varying degrees (Figure 2).

**Figure 3:**
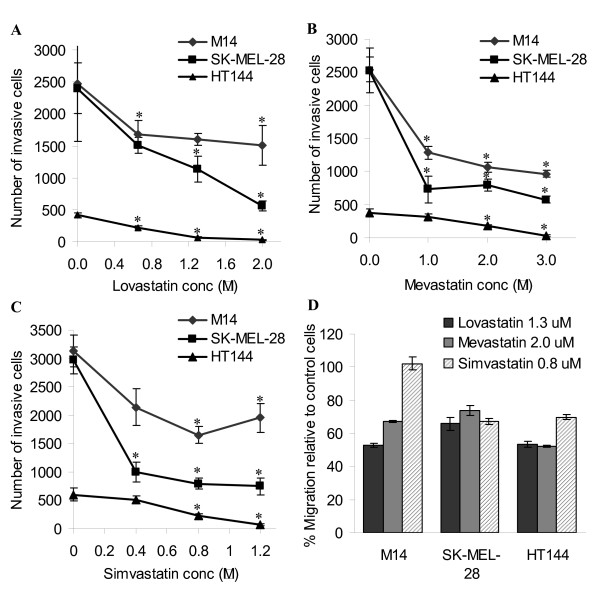
Effects of (A) lovastatin, (B) mevastatin and (C) simvastatin on invasion and (D) migration of melanoma cell lines. 1 × 10^5 ^cells were seeded for each assay. Error bars represent the standard error of the mean for at least 4 assays. * indicates p < 0.05 using the Student's t-test to compare invasion levels of statin treated cells versus control.

To assess whether statins can inhibit melanoma cell invasion, cells were exposed to escalating doses of statin drugs over 24 hours in invasion assays. Growth assays were also performed with these concentrations to ensure that they were non-toxic over the course of the assays. Each of the statin drugs had an inhibitory effect, on invasion, in a dose dependant manner on all three melanoma cell lines (Figure 3A–C). Regression analysis showed a significant trend toward decreased levels of cell invasion with increasing statin concentration in SK-MEL-28 cells (Linear regression: p < 0.001 (lovastatin), p = 0.001 (mevastatin), p < 0.001 (simvastatin)), HT144 cells (Linear regression: p < 0.001 (lovastatin), p < 0.001 (mevastatin), p < 0.001 (simvastatin)), and in M14 cells with the exception of lovastatin (Linear regression: p = 0.073 (lovastatin), p < 0.001 (mevastatin), p = 0.003 (simvastatin)). The effects of statins on cell migration were also examined by exposure of cells in migration assays and the statins inhibited migration in the three melanoma cell lines, with the exception of simvastatin in M14 cells (Figure 3D).

### Effect of simvastatin, lovastatin and mevastatin on adhesion

**Figure 4:**
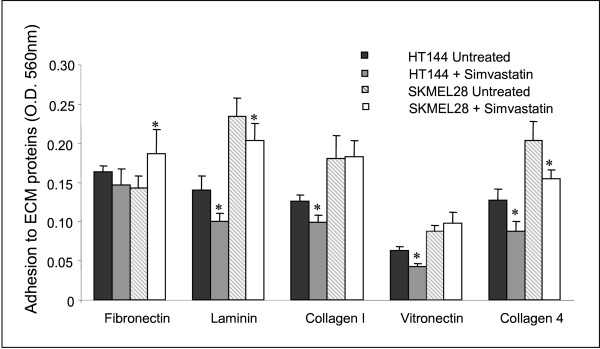
Effect of 0.8 μM simvastatin treatment on adhesion of HT144 and SK-MEL-28 to fibronectin, laminin, collagen type I, vitronectin and collagen type IV. * indicates p < 0.05 using the Student's t-test.

SK-MEL-28 and HT144 melanoma cells were treated with 0.8 μM simvastatin for 24 hours to study the effect of simvastatin on adhesion to the ECM proteins, fibronectin, laminin, collagen type I, vitronectin and collagen type IV. Simvastatin exposure resulted in significantly decreased adhesion to laminin and collagen type IV in both cell lines, while collagen type I and vitronectin adhesion was only significantly decreased in HT144 cells (Figure 4).

**Figure 5:**
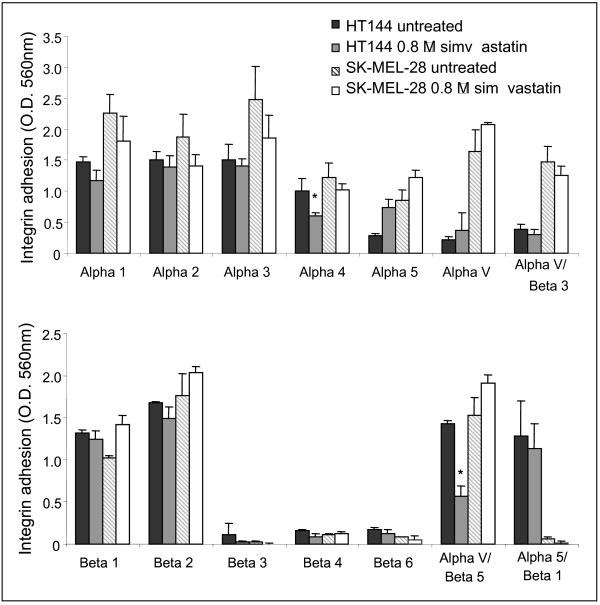
(A) α-Integrin and (B) β-integrin mediated binding profile of untreated HT144, HT144 treated with 0.8 μM simvastatin, SK-MEL-28 untreated and SK-MEL-28 treated with 0.8 μM simvastatin. * indicates p < 0.05 using the Student's t-test.

SK-MEL-28 and HT144 melanoma cells were treated with 0.8 μM simvastatin for 24 hours to study the effect of simvastatin on alpha and beta integrin mediated binding. HT144 displayed significantly decreased alpha 4 and alpha v beta 5 integrin-mediated binding (Figure 5). SK-MEL-28 did not display any significant changes in integrin-mediated binding.

## Discussion

We hypothesized that the statin drugs, simvastatin, lovastatin, mevastatin and pravastatin would have inhibitory effects on melanoma cell proliferation, invasion and cell migration *in vitro*. First we examined the effects of the drugs on cell proliferation in three melanoma, two lung cancer and four breast cancer cell lines. The lipophilic statins, simvastatin, lovastatin and mevastatin inhibited the proliferation of all cell lines tested, while pravastatin, a non-lipophilic statin, did not significantly inhibit proliferation of any of the cell lines tested. This supports previous findings [19,20] and may be explained by the fact that unlike the lipophilic statins, a sodium-independent bile acid transporter mediates uptake of pravastatin by cells, which is absent on most extra-hepatic cells [21]. A significant difference was found in the sensitivity of the melanoma and lung cancer cell lines to statins compared to the breast cancer cell lines in this study. The literature supports these findings, as epidemiological studies have failed to find a link between statin use and breast cancer incidence. Bonovas *et al *[22] performed a meta-analysis of seven large randomised trials and nine observational studies (five case-control and four cohort studies) on breast cancer risk and statin use. Statin use was not found to significantly affect breast cancer risk (fixed effects model: RR = 1.03; 95% CI, 0.93 to 1.14; random effects model: RR = 1.02; 95% CI, 0.89 to 1.18). In addition, Eliassen *et al *[23] retrospectively looked at the associations of statin use and breast cancer risk in the Nurses' Health Study (n = 75,828), with 6 to 12 years of follow-up. 3177 incident cases of invasive breast cancer were documented, but were not associated with statin use (RR, 0.91; 95% CI, 0.76–1.08). A similar lack of association was reported in a recent population based study [24]. These results suggest that statins do not affect the pathology of breast cancer, which may correlate at least in part with our results which show that breast cancer cells are relatively insensitive to statins.

Our findings suggested that melanoma may be sensitive to statin treatment. To further investigate this finding, we examined the effects of statins on melanoma cell apoptosis using the M14 melanoma cell line. Lovastatin, mevastatin and simvastatin increased the percentage of apoptotic cells in a dose-dependant manner. However, the concentrations which induced apoptosis in the M14 cells were significantly higher than the IC_50 _concentrations determined in the proliferation assays. Although the duration of the assays are different, it is probable that the statins have both cytocidal and cytostatic effects in melanoma cells. Lovastatin has previously been reported to induce apoptosis and cell cycle arrest in melanoma cell lines [11,25].

Due to the highly metastatic nature of melanoma, we wanted to determine whether the statin drugs were capable of inhibiting melanoma cell migration and/or invasion. Lovastatin, mevastatin and simvastatin were found to inhibit melanoma cell invasion *in vitro *in all three melanoma cell lines at non-toxic concentrations, in a dose dependant manner. Similar findings were observed in cell migration assays, with the exception of simvastatin in one of the cell lines. These results indicate a dual role for statins in the treatment or prevention of melanoma. Collisson *et al *[14] described atorvastatin inhibition of invasion of the melanoma cell lines A375M, CHL, SK-MEL-28, and WM 166-4 in a dose dependant manner, with A375M invasion being inhibited at non-toxic concentrations. Farina *et al *[26] described the metastatic inhibition of F311 mammary carcinoma cells in BALB/c mice following pre-treatment with a non-cytotoxic concentration of lovastatin.

To further investigate possible mechanisms through which statins may exert their inhibitory effects on invasion and migration of melanoma cells, we examined the effects of simvastatin on the ability of HT144 and SK-MEL-28 to adhere to extracellular matrix proteins. Small changes in adhesion to a number of extracellular matrix proteins were observed. Simvastatin decreased the ability of the cells to adhere to laminin and collagen type IV in both cell lines. Laminin and collagen type IV are important components of the extracellular matrix, and play a role in cell adhesion and motility. Laminin also plays a major role in the remodelling of the melanoma microenvironment, and is required for melanoma vasculogenic mimicry and activation of integrin signalling [27]. Collagen type IV is a regulator of melanoma cell adhesion, proliferation, cell migration and invasion and melanoma induced angiogenesis [28]. Properties distinguishing metastasising and non-metastasising melanoma cells have been described, including an increased capacity for laminin and collagen binding [29]. Thus the inhibition of laminin and collagen type IV cell adhesion by simvastatin, may contribute to its anti-invasive effects.

As extracellular matrix protein adhesion is mediated by integrins we then examined the effects of simvastatin treatment on integrin binding in the melanoma cells. Small changes in integrin binding were also observed but simvastatin treatment significantly decreased the binding of alpha 4 and alpha v beta 5 integrins in HT144 cells, and not in SK-MEL-28. Alpha v beta 5 integrin has been shown to mediate melanoma cell vitronectin binding [30], which is consistent with our findings that simvastatin treatment significantly decreased adhesion of HT144 cells to vitronectin. CNTO 95, an alpha v integrin humanised monoclonal antibody, also inhibits human melanoma cell adhesion, migration and invasion [31]. Alterations in expression of matrix-metalloproteases and cytoskeletal reorganization may also contribute to the effects of the statins on invasion and migration of melanoma cells.

The peak plasma concentration of simvastatin achieved in patients receiving a 40 mg per day dose is approximately 3 ng/ml (7.2 nM) [32]. However, a recent dose finding study in patients with myeloma or lymphoma found that the maximum tolerated dose of simvastatin, given in combination with chemotherapy, was 15 mg/kg/day [33], which suggests that much higher plasma concentrations could be achieved. In a Phase I clinical trial in patients with solid tumours, lovastatin administered at 45 mg/kg/day resulted in peak plasma concentrations of approximately 3.9 μM [34], which is higher than the concentrations required to inhibit proliferation and invasion of melanoma cells *in vitro*.

## Conclusion

Our results suggest that the standard doses used for cholesterol treatment may not be sufficient to directly inhibit melanoma cell proliferation or invasion. Therefore, statins are unlikely to be beneficial for chemoprevention of melanoma. However, statin treatment at higher doses may be beneficial as an adjuvant therapy to inhibit melanoma cell growth, invasion and metastasis. A trial is currently open to examine the effects of lovastatin in treating patients at high risk of recurrent melanoma (NCT00462280 [35]). Synergistic interactions have been observed between statins and several chemotherapy agents *in vitro *[36]. Therefore, it is likely that the greatest potential for statins in melanoma treatment would be in combination with chemotherapy or with emerging targeted therapies. Further research is required to examine whether statins can act either additively or synergistically in combination with chemotherapeutics to increase cell kill, and whether these effects are reversible with the addition of mevalonic acid. Combinations of statins with chemotherapy should first be tested *in vitro *and then proceed to *in vivo *animal studies to examine effects on tumour burden and/or tumour invasion and metastatic spread, prior to initiation of clinical trials examining the addition of high dose statins to chemotherapy in humans.

## Competing interests

The author(s) declare that they have no competing interests.

## Authors' contributions

SG designed the study, performed toxicity testing, invasion and migration assays, adhesion assays, statistical analysis, analysis and interpretation of data, and drafted the manuscript; DOS and AE performed invasion and migration assays; MC co-supervised the study, provided guidance and interpretation of data; NOD performed toxicity testing, apoptosis assays, co-supervised the study and participated in the planning, analysis and interpretation of data, and preparation of the manuscript.

## Pre-publication history

The pre-publication history for this paper can be accessed here:


